# Effectiveness of Rituximab and Its Biosimilar in Treating Adult Steroid-Dependent Minimal Change Disease and Relapse

**DOI:** 10.7759/cureus.49200

**Published:** 2023-11-21

**Authors:** Hui Yi Shan

**Affiliations:** 1 Nephrology and Hypertension, Department of Medicine, Keck School of Medicine of University of Southern California, Los Angeles, USA

**Keywords:** frequent relapses, steroid-dependent minimal change disease, rituximab biosimilar, ruxience, rituximab, minimal change disease

## Abstract

Minimal change disease (MCD) is an important cause of nephrotic syndrome in adults. Its course is often complicated by frequent relapses and steroid dependence. Most of the treatment experience of MCD comes from management of pediatric patients rather than adult patients. In this report, the author describes successful experience of using rituximab (RTX) and its biosimilar, RTX-pvvr (ruxience), to treat steroid-dependent MCD and relapses in adult patients. This is the first report of using a RTX biosimilar to treat MCD. This case series demonstrates that RTX and ruxience are well-tolerated, efficacious treatment for managing adult patients with steroid-dependent MCD and relapses.

## Introduction

Minimal change disease (MCD) is an important cause of nephrotic syndrome in adults, accounting for 10 to 15 percent of cases [1]. While corticosteroid is effective in treating MCD, high relapse rate and steroid dependency have been problematic for patients and challenging for their nephrologists [2]. In addition, long term use of systemic corticosteroids is associated with significant adverse events. Majority of treatment experience for steroid-dependent MCD comes from management of pediatric patients, significantly less is known about the treatment response of MCD in adult patients. In this paper, the author reports successful experience of using rituximab (RTX) and its biosimilar, ruxience, to treat steroid-dependent MCD in adult patients in their 20s and 30s. All patients were able to be tapered off steroid and remained in complete remission for prolonged period. Furthermore, their post-RTX or post-ruxience MCD relapses were treated successfully with small dose and short courses of steroid. This is the first report of using a RTX biosimilar to treat MCD. All patients had three-to-five years of long-term follow up data. With this case series, the author hopes to increase awareness in using RTX and its biosimilar, ruxience, as effective treatments for managing steroid-dependent MCD and relapses in adults.

## Case presentation

Case 1

**Figure 1:**
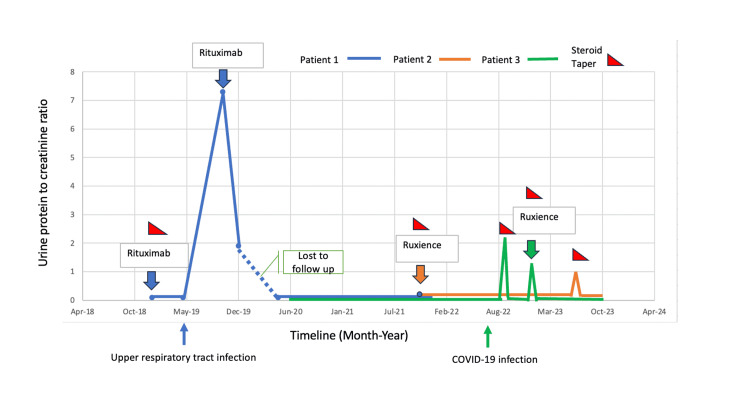
Proteinuria trend with RTX or ruxience treatment RTX: Rituximab

A 28-year-old female was diagnosed with MCD with renal biopsy in 2015. The patient was treated with prednisone but was unable to taper prednisone to dose below 20 mg daily without experiencing a relapse. She came to clinic for a second opinion in January 2019. After a negative tuberculosis and hepatitis B screening, the patient received RTX 375 mg/m^2^, four-weekly infusions. Her post-infusion absolute CD 19 positive cell count was 0 x 10^6^/L. Prednisone was subsequently tapered off in six weeks. She stayed in complete remission until October 2019 when she experienced a relapse of MCD, with proteinuria reaching 7.3 g and serum albumin of 3 g/dL, in the setting of a recent upper respiratory tract infection. At that time, her absolute CD 19 positive cell count was 26 x 10^6^/L (normal range is 91-295 x 10^6^/L). A second course of RTX was given (1 g on Day 1 and Day 15). Her proteinuria went from 7.3 g to 1.9 g in two months. The patient was lost to follow up for one year due to travel and returned in May 2021 with urine protein/creatinine 0.2 mg/mg. Her absolute CD 19 positive cell count at that time was 78 x 10^6^/L. No additional immunosuppression regimen was given in the interim (Figure 1). The patient stayed in complete remission until January 2022 when she contracted COVID-19. Her proteinuria increased, as noted by positive protein in urinalysis. Urine protein/creatinine was not performed. She was treated with prednisone 10 mg daily and tapered off in one month. She has since stayed in remission for close to two years.

Case 2

A 34-year-old male was diagnosed with MCD with renal biopsy in 2008. His MCD was treated with prednisone and went into complete remission. Since 2008, the patient experienced four relapses. Each time, he received a course of prednisone. His last relapse was in 2018. Since then, he has been maintained on 10 mg prednisone chronically and was not able to get off prednisone without experiencing an increase in proteinuria. The patient’s bone density test showed osteopenia in the left femoral neck with a T-score of -1.3. He came for a second opinion in August 2021 due to concern of prolonged steroid use and developing a side effect of osteopenia at a young age. This patient was treated with ruxience 1 g IV on Day 1 and Day 15. Prednisone 10 mg daily was subsequently tapered off after two months. He remained in complete remission for 23 months until July 2023, when his urine protein to creatinine ratio increased from 0.06 to 0.9 mg/mg. Because his relapse was detected early and proteinuria was mild, the patient was treated with prednisone 20mg daily; a repeat urine protein to creatinine ratio was below detectable range 20 days later. The patient was then tapered off prednisone over a span of six weeks and remained in complete remission (Figure 1).

Case 3

A 31-year-old male was diagnosed with MCD with renal biopsy at age 13. He had a history of multiple relapses and was maintained on steroid and cyclosporine for more than 13 years. The patient self-stopped immunosuppression in 2019 during COVID pandemic. In June 2022, he contracted COVID-19 infection and his MCD relapsed two months later with urine protein to creatinine ratio of 2.3 mg/mg. The patient was treated with 60 mg of prednisone daily and gradual taper of steroid dosage, with improvement of proteinuria. However, he relapsed again in December 2022, urine protein went up to 1.2 g. He was started on prednisone followed by ruxience 1 g IV x two doses on Day 1 and Day 15, which he completed on January 9, 2023. Urine protein to creatinine ratio normalized to 0.1 mg/mg, and prednisone was tapered off over two months. At four months post-infusion, his absolute CD 19 positive cell count was checked and it remained at 0 x 10^6^/L.The patient has stayed in complete remission for more than 10 months until present (Figure 1).

## Discussion

Steroid-dependent MCD and relapses are challenging to treat. Patients with steroid dependence and frequent relapses suffer from well-known side effects of long-term corticosteroids use. Glucocorticoid (GC)-associated toxicity appears to be related to both the average dose and cumulative duration of GC use. These adverse events include osteoporosis and fractures, adrenal suppression, hyperglycemia and diabetes, cushingoid appearance and weight gain, cardiovascular disease, psychiatric disturbances, immunosuppression, and dermatological changes [3].

Rituximab (RTX), a monoclonal antibody against CD20-bearing cells, depletes CD20+ B lymphocytes and showed satisfactory safety and efficacy profile in pediatric nephrotic syndrome patients [4]. The cases described in this report demonstrated the effectiveness of RTX and its biosimilar, ruxience (rituximab-pvvr), in treating MCD relapse and steroid dependency in adult patients. Patients 1 and 2 spent long years of being on maintenance prednisone. They were successfully tapered off prednisone after receiving RTX or ruxience infusion. In addition, both patients were able to maintain sustained complete remission. Patient 1’s longest remission period was 27 months, and Patient 2 was in complete remission for 23 months. This supports the efficacy of RTX or ruxience in treating steroid-dependent MCD as monotherapy. Xue et al. published a meta-analysis by pooling data of cohort studies and case series on adult patients with frequent relapsing or steroid-dependent MCD or focal segmental glomerulosclerosis (FSGS). The study found a pooled rate of complete remission after RTX treatment was 84.2% and that of partial remission was 5.8%. The complete remission rates were 100% in Asians and 69.6% in Caucasians. Notably, pooled comparison of the number of relapses per year before and after RTX treatment showed that the relapse rate significantly decreased by 2.15 times/year; the rate of relapse post-RTX treatment was 27.4% adjusted by sample size. Furthermore, RTX also significantly reduced the steroid dose by 17.8mg/day [5].

This paper is the first report of using RTX biosimilar in treating steroid-dependent MCD. Ruxience was approved by the US Food and Drug Administration (FDA) in 2019 for the treatment of non-Hodgkin's lymphoma, chronic lymphocytic leukemia, granulomatosis with polyangiitis and microscopic polyangiitis [6]. There are also other RTX biosimilar available in the market that include truxima and riabni [7].

For Patients 2 and 3, ruxience was used instead of RTX due to insurance coverage. In both patients, ruxience was effectiveness in treating MCD. The average cost of RTX is $99,900/g versus $76,400/g of ruxience [8,9]. This leads to a difference of $47,000 for each course of treatment, using the 1 g on Day 1 and Day 15 regimen. The RTX biosimilar provides a significantly cost-saving alternative for patients with MCD. Both patients tolerated ruxience infusion well without significant short-term or long-term adverse events. Another important and novel observation was that once patients received RTX or ruxience, a short course of low dose prednisone (20 mg or less per day for four-eight weeks) was able to successfully treat their subsequent relapses. 

Respiratory tract infection has been reported to be linked to adult-onset and relapse of MCD. In a study published by Han et al., 87 patients with incipient MCD were enrolled, the study found that before disease onset, 20.7% (18/87) of patients with incipient MCD were diagnosed with infection, including 94.5% (17/18) with respiratory tract infection. 14 patients in complete remission post treatment developed an infection before relapse, including 85% (12/14) with respiratory tract infection [10]. Two of the three patients described in this report had relapse of MCD after respiratory tract infections. Specifically, they developed MCD relapse after contracting COVID-19 infections. While kidney injury is one of the known complications following COVID-19 infection and vaccination, only few cases of MCD following COVID-19 infection and vaccination have been reported [11-13]. Our patient cases expand the clinicians’ experience in treating MCD relapses following COVID-19 infection. Further studies are needed to determine the incidence and pathophysiology of MCD either post COVID-19 vaccines or following COVID-19 infections.

## Conclusions

This report illustrates that RTX and its biosimilar, ruxience, are well-tolerated, efficacious treatments for managing adult patients with steroid-dependent MCD and relapses. One course of treatment provides long-lasting effect to allow successful steroid withdrawal and sustained complete remission of disease. Importantly, these treatments significantly lessen the need for long-term, high-dose corticosteroid use and, hence, minimize the GC-associated toxicity in these patients.
